# COVID‐19 rapid antigen tests approved for self‐testing in Australia: published diagnostic test accuracy studies and manufacturer‐supplied information. A systematic review

**DOI:** 10.5694/mja2.52151

**Published:** 2023-10-30

**Authors:** Katy JL Bell, Yuyang Li, Ellie Medcalf, Deonna Ackermann

**Affiliations:** ^1^ School of Public Health the University of Sydney Sydney NSW

**Keywords:** COVID‐19, Diagnostic tests and procedures, Public health, Evidence‐based medicine, Systematic review

## Abstract

**Objectives:**

To review evaluations of the diagnostic accuracy of coronavirus disease 2019 (COVID‐19) rapid antigen tests (RATs) approved by the Therapeutic Goods Administration (TGA) for self‐testing by ambulatory people in Australia; to compare these estimates with values reported by test manufacturers.

**Study design:**

Systematic review of publications in any language that reported cross‐sectional, case–control, or cohort studies in which the participants were ambulatory people in the community or health care workers in hospitals in whom severe acute respiratory syndrome coronavirus 2 (SARS‐CoV‐2) infection was suspected, and the results of testing self‐collected biological samples with a TGA‐approved COVID‐19 RAT were compared with those of reverse transcription–polymerase chain reaction (RT‐PCR) testing for SARS‐CoV‐2. Estimates of diagnostic accuracy (sensitivity, specificity) were checked and compared with manufacturer estimates published on the TGA website.

**Data sources:**

Publications (to 1 September 2022) identified in the Cochrane COVID‐19 Study Register and the World Health Organization COVID‐19 research database. Information on manufacturer diagnostic accuracy evaluations was obtained from the TGA website.

**Data synthesis:**

Twelve publications that reported a total of eighteen evaluations of eight RATs approved by the TGA for self‐testing (manufacturers: All Test, Roche, Flowflex, MP Biomedicals, Clungene, Panbio, V‐Chek, Whistling) were identified. Five studies were undertaken in the Netherlands, two each in Germany and the United States, and one each in Denmark, Belgium, and Canada; test sample collection was unsupervised in twelve studies, and supervised by health care workers or researchers in six. Estimated sensitivity with unsupervised sample collection ranged from 20.9% (MP Biomedicals) to 74.3% (Roche), and with supervised collection from 7.7% (V‐Chek) to 84.4% (Panbio); the estimates were between 8.2 and 88 percentage points lower than the values reported by the manufacturers. Test specificity was high for all RATs (97.9–100%).

**Conclusions:**

The risk of false negative results when using COVID‐19 RATs for self‐testing may be considerably higher than apparent in manufacturer reports on the TGA website, with implications for the reliability of these tests for ruling out infection.

The World Health Organization (WHO) has noted that testing for the severe acute respiratory syndrome coronavirus 2 (SARS‐CoV‐2) continues to support global efforts to reduce the morbidity and mortality associated with coronavirus disease 2019 (COVID‐19) by facilitating timely care and treatment and reducing viral transmission.^1^ Reverse transcription polymerase chain reaction (RT‐PCR) testing is the reference standard for detecting SARS‐CoV‐2 infections.^2^ However, as RT‐PCR testing in Australia now requires a referral from a general practitioner or nurse practitioner,^3^ self‐testing using rapid antigen tests (RATs) has become the main method for identifying SARS‐CoV‐2 infections.^4^ RATs are readily available from pharmacies, retail outlets, and online suppliers.

In Australia, the Therapeutic Goods Administration (TGA) first approved RATs for self‐testing in November 2021.^5^ By 1 September 2022, 53 RATs had been approved, each with an entry in the Australian Register of Therapeutic Goods,^6^ classified by the TGA according to the estimated sensitivity reported by the manufacturer: “acceptable sensitivity” (greater than 80%), “high sensitivity” (greater than 90%), and “very high sensitivity” (greater than 95%).^5^ However, sensitivity estimates provided by manufacturers may not reflect performance when test kits are used for self‐testing at home.^2^

For this review, we systematically collated and appraised published evaluations, based upon confirmatory RT‐PCR testing for SARS‐CoV‐2 infections, of the diagnostic accuracy of COVID‐19 RATs approved by the TGA for self‐testing by ambulatory people. We then compared these diagnostic accuracy estimates with manufacturer estimates published on the TGA website.

## Methods

### Information sources and search strategy

We searched for publications to 1 September 2022 in the Cochrane COVID‐19 Study Register^7^ and the WHO COVID‐19 research database.^8^ The two databases are living data repositories, regularly updated by searches of MEDLINE, EMBASE, the bioRxiv and medRxiv preprint servers, and several other databases^9^, ^10^ using specific search strategies developed by information science specialists.^7^, ^8^ We report our review according to the PRISMA 2020 guidelines.^11^

### Publication eligibility criteria

To identify potentially relevant records in the two COVID‐19 research repositories, we searched for the following terms: COVID‐19 testing, SARS‐CoV‐2 testing, rapid antigen test*, self‐test*, sensitivity and specificity, diagnostic accuracy, diagnostic performance, and the names of each of the 53 TGA‐approved RATs.

We included publications in any language that described cross‐sectional, case–control, or cohort studies in which the participants were ambulatory people in the community or health care workers in hospitals with suspected SARS‐CoV‐2 infections, and the results of testing self‐collected biological samples with a TGA‐approved COVID‐19 RAT (index test) were compared with those of RT‐PCR testing (reference standard) for SARS‐CoV‐2 infection (target condition).

We excluded publications that reported retrospective studies, and those in which participant selection was not clearly described, the index test was not performed at the time of sample collection, the RAT samples were not collected by the tested person themselves, the sample type or collection method did not comply with the manufacturer's instructions, or the diagnostic accuracy data were not reported or were not adequate for sensitivity and specificity calculations. We also excluded reviews, opinion articles, editorials, and letters that did not include original data, and articles for which the full text was not available.

### Study selection and screening

All records identified in the database searches were collated and uploaded to Covidence (Veritas Health Innovation) and duplicates removed. One reviewer (author YL) screened titles and abstracts according to our eligibility criteria. The full text of potentially relevant records was then assessed by one reviewer (YL) according to the eligibility criteria and checked by a second reviewer (KB, EM, or DA); discrepancies were resolved by discussion by the full team.

We downloaded from the TGA database^5^ the user instructions and clinical data supplied by the manufacturers for the COVID‐19 RATs used in the studies that satisfied our inclusion criteria.

### Data extraction

We used a standardised form for extracting information on the characteristics and outcomes of the included studies: author, publication year, country, study design, setting, participant number and characteristics, participant symptoms, RAT brand, test sample type, supervision of sample collection, number of RT‐PCR‐positive participants, reported sensitivity and specificity, and numbers of true positive, false positive, true negative, and false negative test results. Data extraction was undertaken by one reviewer (KB, YL, EM, or DA) and checked by a second (KB, YL, EM, or DA).

### Assessment of risk of bias

One reviewer (KB, YL, EM, or DA) assessed the risk of bias (domains: participant characteristics, index test, reference test, flow and timing of tests) for each study using the QUADAS‐2 tool;^12^ a second reviewer checked each assessment. Disagreements were resolved by consensus.

### Analysis

The principal diagnostic accuracy measure for this study was sensitivity per person. We calculated sensitivity and specificity for each study from the reported numbers of true positive, false positive, false negative, and true negative test results to check the sensitivity and specificity reported by the study authors. We then compared the publication values with those reported by the respective manufacturers and published on the TGA website.

We plotted paired sensitivity and specificity estimates for each study in a forest plot using the R package *DTAplots*;^13^ the PRISMA flowchart was generated with the *PRISMA2020* Shiny app;^14^ and the risk of bias assessment figures were constructed with the *robvis* Shiny app.^15^

### Ethics approval

For this negligible risk research study we analysed publicly available, non‐identifiable aggregated data, and our investigation was therefore exempt from formal ethics review. The review protocol was not registered.

## Results

A total of 1842 unique potentially relevant records were identified by the Cochrane and WHO database searches. The full text of 296 articles was screened, of which 284 were excluded (Supporting Information, figure 1). Twelve studies were included in our review, all in English: ten peer‐reviewed journal articles^17^, ^18^, ^20^, ^21^, ^22^, ^23^, ^24^, ^25^, ^26^, ^27^ and two preprints^16^, ^19^ (Box 1).

Box 1 **Characteristics of studies of Australian Therapeutic Goods Authority‐approved COVID‐19 rapid antigen self‐tests included in our systematic analysis**

**Table:** 

Author (publication year), country	Study design and setting	Participant characteristics and symptoms*	Rapid antigen test
**Unsupervised sample collection**			
Zwart (2022),^16^ Netherlands	Prospective cross‐sectional diagnostic test accuracy study of health care workers presenting for routine SARS‐CoV‐2 PCR testing at a hospital clinic, 31 Oct 2020 and 2 Feb 2021 Health care workers in secondary care hospitals, academic teaching hospitals, and long term care facilities. Alpha variant circulating; Delta or Omicron variants of SARS‐CoV‐2 had not yet been detected in the Netherlands	Age: median, 40 years (interquartile range, 30–52 years) Women: 83.1% Medical professionals: 78.5% Exposure history: not provided Symptoms at time of sampling: 77.1% (no further details)	Roche SARS‐CoV‐2 Rapid Antigen Test (oropharyngeal and nasal)
Stohr (2022),^17^ Netherlands	Prospective cross‐sectional diagnostic test accuracy study of people presenting for routine SARS‐CoV‐2 PCR testing at public health service test sites, 23 Dec 2020 – 17 Jan 2021 Dutch public health service test site in Tilburg, North Brabant	Age: median, 41 years Women: 57.2% Level of education: 48.5% high school, 34.8% bachelor's degree Exposure history: not provided Symptoms of COVID‐19 at time of testing: 68.6% No symptoms of COVID‐19 in past three weeks: 93.6%	Roche SARS‐CoV‐2 Antigen Self‐Test (nasal)
Møller (2022),^18^ Denmark	Prospective cross‐sectional diagnostic accuracy study of people presenting for routine SARS‐CoV‐2 PCR testing at a testing centre, 21 and 25 Jan 2021 University Hospital in Aarhus. At the time of the study, 11–16% of population underwent PCR testing (0.1–0.6% positive)	Age: mean, 42 years Women: 50.5% Health professionals: 14.6% No vaccine doses: 95.4% Previous COVID‐19: none Exposure history: close contact with confirmed case, 13% Symptoms on PCR test day: 17%	Panbio COVID‐19 Antigen Rapid Test Device (nasal)
Iftner (2022),^19^ Germany	Prospective cross‐sectional diagnostic test accuracy study of asymptomatic employees of a university hospital, 12 May 2021 – 20 July 2021 University Hospital, Tübingen, Germany Low prevalence of COVID‐19 at time of the study	No details provided, except probably high number of medical staff None had symptoms	Clungene COVID‐19 Antigen Rapid Test (nasal, researcher interpretation)
			Clungene COVID‐19 Antigen Rapid Test (nasal; participant interpretation)
Schuit (2022),^20^ Netherlands	Prospective cross‐sectional diagnostic test accuracy study of people presenting for routine SARS‐CoV‐2 PCR testing, 9–26 Sept 2021 Public health service test sites in West Brabant (Roosendaal), Central and Northeast Brabant (Tilburg), and Rotterdam‐Rijnmond (Zuidland) SARS‐CoV‐2 prevalence (Netherlands): 8.2% (99.9% Delta variant)	Age: mean, 40.9 years Women: 61.3% Two vaccine doses: 74.2% At least one prior SARS‐CoV‐2 infection: 13.1% Exposure history: asymptomatic and close contact of confirmed infected person, 9% Symptoms at time of sampling: 82.7% (cold symptoms, 91.0%; shortness of breath, 16.7%; fever, 18.0%; coughing, 54.1%) Symptoms within one day of sampling: 48.1%	All Test COVID‐19 Antigen Rapid Test (oral fluid)
			Roche SARS‐CoV‐2 Antigen Self‐Test (nasal)
Schuit (2022),^21^ Netherlands	Prospective cross‐sectional diagnostic test accuracy study of people presenting for routine SARS‐CoV‐2 PCR testing at public health service test sites, 21 Dec 2021 – 10 Feb 2022 Dutch public health service test sites in Rotterdam‐Rijnmond (Rotterdam), Central and Northeast Brabant (Tilburg), and West‐Brabant (Roosendaal), Netherlands. Omicron variant prevalence: 29% (week 51 of 2021), 99% (week 5 of 2022; BA.1 variant: > 95%); from 12 Jan 2022: Omicron > 90% of infections	Age: mean, 37 years (range, 16–77 years) Women: 57.6% Three vaccine doses: 50.3% At least one prior SARS‐CoV‐2 infection: 23.9% Exposure history: close contact with confirmed case: 11.6% All participants had symptoms suggesting SARS‐CoV‐2 (cold symptoms, 87.6%; shortness of breath, 15.0%; fever, 23.1%; cough 50.0%)	Flowflex SARS‐CoV‐2 Antigen Rapid Test (nasal)
			MP Biomedicals Rapid SARS‐COV‐2 Antigen Test Card (nasal)
			MP Biomedicals Rapid SARS‐COV‐2 Antigen Test Card (oropharyngeal and nasal)
Venekamp (2023),^22^ Netherlands	Prospective cross‐sectional diagnostic test accuracy study of people presenting for routine SARS‐CoV‐2 PCR testing at public health service test sites, 12 Jan – 30 Mar 2022 Dutch public health service test sites in Rotterdam and Tilburg Omicron variant prevalence: > 90% of circulating SARS‐CoV‐2 on 12 January, > 99.5% from 31 January	Age: mean, 39 years (range, 16–80 years) Women: 53.3% Three vaccine doses: 62.4% At least one prior SARS‐CoV‐2 infection: 29.9% Exposure history: close contact as reason for testing: 84.2% No participants had symptoms suggesting SARS‐CoV‐2 infection; 3.3% reported symptoms as reason for testing	Flowflex SARS‐CoV‐2 Antigen Rapid Test (nasal)
			MP Biomedicals Rapid SARS‐COV‐2 Antigen Test Card (nasal)
**Supervised sample collection**			
Shah (2021),^23^ USA	Prospective cross‐sectional diagnostic study of unvaccinated registrants at a community SARS‐CoV‐2 testing site, 16 Nov 2020 – 15 Dec 2020 Oshkosh, Wisconsin SARS‐CoV‐2 infection prevalence, 15.8% (variant not reported)	Age: < 18 years, 10.7% Women: 56.1% White: 95% Exposure history: within past 14 days, 42.4% Symptoms: 56.3%	BinaxNOW COVID‐19 Antigen Card Self‐Test^†^ (nasal)
Klein (2021),^24^ Germany	Prospective cross‐sectional diagnostic test accuracy study of people presenting for routine SARS‐CoV‐2 PCR testing at a German drive‐in testing centre, 15 Dec 2020 – 19 Jan 2021 Drive‐in test centre, led by local health authority in Heidelberg	Age: mean, 42.7 years (SD, 14.6 years) Women: 52.4% Comorbidity: 33.8% Exposure history: not provided Symptoms on day of testing: 45.9% Mean duration of symptoms: 3.8 days (SD, 5.4 days)	Panbio COVID‐19 Antigen Rapid Test Device (nasopharyngeal)
DeMeyer (2022),^25^ Belgium	Prospective cross‐sectional diagnostic test accuracy study of people (10% children) presenting for routine SARS‐CoV‐2 PCR testing at a testing centre, 16 Dec 2021 – 7 Jan 2022 University hospital outpatient testing in Antwerp Omicron BA.1 variant emerging in Belgium	Age: median, 31 (Whistling) or 39 years (V‐Chek); range, 7–72 years Women: 56.1% Exposure history: not provided Participants met national testing strategy guidelines, but details on symptoms not provided	V‐Chek COVID‐19 Antigen Saliva Test (oral)
			Whistling test 2019‐nCoV Saliva Ag Easy Test (oral)
Goodall (2022),^26^ Canada	Prospective cross‐sectional diagnostic test accuracy study of people who did not report symptoms attending an urban rapid testing centre, seven days in January 2022 Nova Scotia, Canada	No details provided Clinic is for people without symptoms	Panbio COVID‐19 Antigen Rapid Test Device (nasal)
Landaverde (2022),^27^ USA	Prospective cross‐sectional diagnostic test accuracy study of people attending a testing site at a university in the USA, 7–11 and 14–17 Feb 2022 University campus testing site in Boston Omicron variant predominant	No details provided Clinic is for people with symptoms, close contacts, or previously positive people scheduled for follow up testing	BinaxNOW COVID‐19 Antigen Card Self‐Test^†^ (nasal)

COVID‐19 = coronavirus disease 2019; PCR = polymerase chain reaction; SARS‐CoV‐2 = severe acute respiratory syndrome coronavirus; SD = standard deviation; USA = United States of America.

* Characteristics and symptoms for Schuit^20^ refer to All Test evaluation (similar for Roche evaluation); for Venekemp^22^ to Flowflex evaluation (similar for MP Biomedicals evaluation); for Schuit^21^ to Flowflex evaluation (similar for MP Biomedicals evaluations) and for all participants, including those undertaking confirmatory testing after prior positive self‐test (characteristics of non‐confirmatory testers not provided); for Møller^18^ to the overall study, which included other tests not approved by the TGA (characteristics for the Panbio evaluation not provided). Characteristics for De Meyer^25^ refer to V‐Chek evaluation (similar for Whistling evaluation).

† BinaxNOW COVID‐19 Antigen Card Self‐Test, the Abbott test marketed in the USA, is identical to the Panbio COVID‐19 Antigen Rapid Test Device.

The twelve included studies reported eighteen evaluations of RATs in a total of 18 430 participants. Tests by eight manufacturers of TGA‐approved COVID‐19 RATs were evaluated: All Test,^20^ Roche,^16^, ^17^, ^20^ Flowflex,^21^, ^22^ MP Biomedicals,^21^, ^22^ Clungene,^19^ Panbio^18^, ^23^, ^24^, ^26^, ^27^ (two:^23^, ^27^ BinaxNOW, the United States name for the Panbio test), V‐Chek,^25^ and Whistling.^25^ No eligible study was identified for 45 of the 53 TGA‐approved tests. None of the included studies were funded by test manufacturers. The authors of one study declared potential financial conflicts of interest,^24^ while the authors of eleven studies declared no potential conflicts of interest. Five studies were undertaken in the Netherlands,^16^, ^17^, ^20^, ^21^, ^22^ two each in Germany^19^, ^24^ and the United States,^23^, ^27^ and one each in Denmark,^18^ Belgium,^25^ and Canada^26^ (Box 1).

Sample collection was unsupervised in twelve studies;^16^, ^17^, ^18^, ^19^, ^20^, ^21^, ^22^, ^23^, ^24^, ^25^, ^26^, ^27^ in six studies sample collection supervised by health care workers or researchers.^23^, ^24^, ^25^, ^26^, ^27^ The sample sizes for the eighteen evaluations ranged from 50 to 2819 participants, the mean or median age from 31 to 41 years, and the proportion of women from 50.5% to 83%. In one study, the participants did not have symptoms suggesting SARS‐CoV‐2 infection,^22^ and in two studies participants did not report any symptoms;^19^, ^26^ in the other evaluations, most participants had symptoms consistent with COVID‐19 or had been in close contact with someone with a confirmed SARS‐CoV‐2 infection. In the studies that reported rates of vaccination and prior COVID‐19, they differed according to study date; for example, 94.5% of participants in the January 2021 Danish study had not received a vaccine dose,^18^ whereas more than 50% of participants in the Dutch study undertaken in early 2022 had received three vaccine doses^22^ (Box 1).

### Risk of bias: published studies

The risk of bias in the participant characteristics domain was low for five studies;^16^, ^17^, ^20^, ^21^, ^22^ it was high for one study because it included participants known to be SARS‐CoV‐2‐positive,^27^ and possibly inappropriate exclusions and inclusions led to concerns about this domain for six studies.^18^, ^19^, ^23^, ^24^, ^25^, ^26^ The risk of bias for the index test was low for all but one study, for which concerns were raised by the possibility that participants were not blinded to their RT‐PCR reference test results at the time of the RAT.^18^ Risk of bias for the reference test was low for nine studies; for three studies,^24^, ^25^, ^26^ concerns were related to RATs being conducted under the supervision of health care workers or researchers in hospitals, so that index test results may have been known to the laboratory undertaking the RT‐PCR (ie, reference test operators were not blinded to index test results). The risk of bias for flow and timing of tests was low for ten studies; the risk was high in one study because of the large proportion of invalid test results (excluded from the analysis),^25^ and for a second study because some index tests were undertaken as long as 72 hours after sample collection for the reference test^18^ (Supporting Information, figure 2).

### Risk of bias: manufacturer‐supplied information

Manufacturers provided no information about participant selection in six studies; for two studies at high risk of bias in this domain, we deemed a case–control design or non‐consecutive recruitment likely, as about half the participants in each study were SARS‐CoV‐2‐positive. For the index test domain, risk of bias was low for the Roche study, and seven manufacturers provided no information about the index test. For the reference standard domain, the risk of bias was high for the Panbio study because the reference standard was a RAT version not intended for self‐use; seven manufacturers provided no information about the reference standard. For the flow and timing domain, risk of bias was low for the Roche study and high for the VChek study (results were reported per sample rather than per person); six manufacturers provided no information for this domain (Supporting Information, figure 3).

### Diagnostic accuracy

Estimated sensitivity with unsupervised sample collection ranged from 20.9% (MP Biomedicals)^22^ to 74.3% (Roche),^16^ and with supervised collection from 7.7% (V‐Chek)^25^ to 84.4% (Panbio).^24^ The estimates were between 8.2 and 88 percentage points lower than the sensitivity values reported by the manufacturer and published on the TGA website (Box 2). Our calculations of test sensitivity from data supplied in the published studies concurred with those reported in the studies (Box 3).

Box 2 **Therapeutic Goods Authority (TGA)‐approved SARS‐CoV‐2 rapid antigen tests for self‐testing: sensitivity as reported in published studies and by test manufacturers**

**Table:** 

Author (publication year), country	Rapid antigen test (RAT)	Sample size*	RT‐PCR‐positive	Sensitivity reported by study	Sensitivity reported by manufacturer	Difference (percentage points)
**Unsupervised sample collection**						
Zwart (2022),^16^ Netherlands	Roche SARS‐CoV‐2 Rapid Antigen Test (oropharyngeal and nasal)	2192	152	74.3%	82.5%	8.2
Stohr (2022),^17^ Netherlands	Roche SARS‐CoV‐2 Antigen Self‐Test (nasal)	1583	192	61.5%	82.5%	21.0
Møller (2022),^18^ Denmark	Panbio COVID‐19 Antigen Rapid Test Device (nasal)	388	0	NA	95.2%	NA
Iftner (2022),^19^ Germany	Clungene COVID‐19 Antigen Rapid Test (nasal, researcher interpretation)	478	0	NA	95.1%	NA
	Clungene COVID‐19 Antigen Rapid Test (nasal, participant interpretation)	476	0	NA	95.1%	NA
Schuit (2022),^20^ Netherlands	All Test COVID‐19 Antigen Rapid Test (oral fluid)	2803	182	46.7%	90.1%	43.4
	Roche SARS‐CoV‐2 Antigen Self‐Test (nasal)	2819	180	68.9%	82.5%	13.6
Schuit (2022),^21^ Netherlands	Flowflex SARS‐CoV‐2 Antigen Rapid Test (nasal)	341	145	52.4%	97.1%	44.7
	MP Biomedicals Rapid SARS‐COV‐2 Antigen Test Card (nasal)	581	169	51.5%	98.2%	46.7
	MP Biomedicals Rapid SARS‐COV‐2 Antigen Test Card (oropharyngeal and nasal)	255	88	69.3%	98.2%	28.9
Venekamp (2023),^22^ Netherlands	Flowflex SARS‐CoV‐2 Antigen Rapid Test (nasal)	1229	193	27.5%	97.1%	69.6
	MP Biomedicals Rapid SARS‐COV‐2 Antigen Test Card (nasal)	1027	115	20.9%	82.5%	61.6
**Supervised sample collection**						
Shah (2021),^23^ USA	BinaxNOW COVID‐19 Antigen Card Self‐Test (nasal)	2110	334	77.2%	95.2%^†^	18.0
Klein (2021),^24^ Germany	Panbio COVID‐19 Antigen Rapid Test Device (nasopharyngeal)	290	45	84.4%	95.2%	10.8
DeMeyer (2022),^25^ Belgium	V‐Chek COVID‐19 Antigen Saliva Test (oral)	50	13	7.7%	95.7%	88.0
	Whistling test 2019‐nCoV Saliva Ag Easy Test (oral)	102	55	9.1%	93.9%	84.8
Goodall (2022),^26^ Canada	Panbio COVID‐19 Antigen Rapid Test Device (nasal)	825	62	64.5%	95.2%	30.7
Landaverde (2022),^27^ USA	BinaxNOW COVID‐19 Antigen Card Self‐Test^‡^ (nasal)	209	54	55.6%	95.2%^†^	39.6

COVID‐19 = coronavirus disease 2019; NA = not applicable; RT‐PCR = reverse transcription–polymerase chain reaction SARS‐CoV‐2 = severe acute respiratory syndrome coronavirus; USA = United States of America.

* Does not include participants for whom RAT or RT‐PCR results were unclear or inconclusive.

† Manufacturer‐reported sensitivity is for Panbio COVID‐19 Antigen Rapid Test Device.

Box 3 **Therapeutic Goods Authority (TGA)‐approved SARS‐CoV‐2 rapid antigen tests for self‐testing: sensitivity and specificity as estimated from data included in published studies**

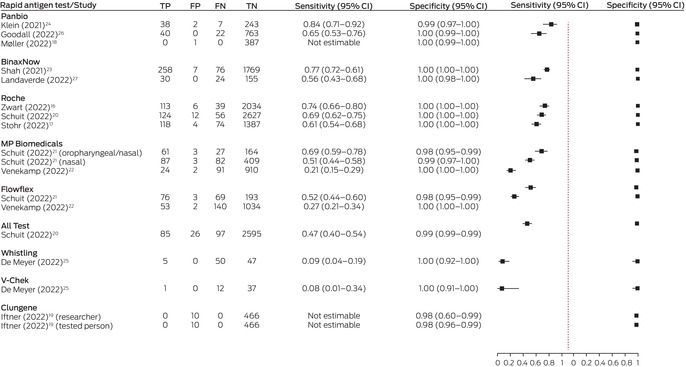

TP = true positive; FP = false positive; FN = false negative; TN = true negative.

Four studies directly compared different TGA‐approved tests, including three in the Dutch public testing system and at low risk of bias across all domains. In two of the Dutch studies, the participants were people with COVID‐19 symptoms: in the first study, the sensitivity of the Roche SD Biosensor nasal self‐test (68.9%) was higher than that of the All Test oral self‐test (46.7%);^20^ in the second the sensitivity of the MP Biomedicals self‐test with combined oropharyngeal and nasal sampling (69.3%) was higher than that of the MP Biomedicals (51.5%) and Flowflex nasal self‐tests (52.4%).^21^ The third Dutch study, in which participants did not have COVID‐19 symptoms, the sensitivity of the Flowflex (27.5%) and the MP Biomedicals nasal self‐tests (20.9%) was similarly low.^22^ The fourth study that compared TGA‐approved tests, in a Belgian university hospital outpatient testing clinic, found that the sensitivity of both the V‐Chek (7.7%) and Whistling saliva tests (9.1%), with supervised sample collection, was very low.^25^ Sensitivity could not be estimated in the two studies in which no participants were SARS‐CoV‐2‐positive by RT‐PCR.^18^, ^19^

Estimated specificity was high for all tests (97.9–100%) and within 2.5 percentage points of the values reported by manufacturers (Supporting Information, table).

## Discussion

At the time of our literature search (1 September 2022), the TGA had approved 53 COVID‐19 RATs for self‐use, of which eight had been evaluated in the twelve studies included in our review, including one test (Whistling) for which TGA approval has since been revoked. Further, only seven of the twelve studies included in our review (evaluations of six TGA‐approved RATs) were based upon unsupervised sample collection and interpretation. No relevant studies have been undertaken in Australia, despite the extremely high rates of COVID‐19 testing in this country during 2020–22;^28^ comparative diagnostic accuracy studies in the public testing system could have provided valuable local evidence regarding RATs approved or under consideration by the TGA. The scarcity of robust evidence for the diagnostic accuracy of these tests when used as intended (ie, without supervision by medical personnel) is striking.

Estimated sensitivity varied substantially between tests and studies and were (often substantially) lower than the manufacturer‐reported values on the TGA website. The consequence is that the risk of false negative results is high when these tests are used for self‐testing at home, a problem unlikely to be appreciated by people who inform themselves on the TGA website about test performance. Decisions based on false negative results could hamper control of SARS‐CoV‐2 transmission, as infected people may decide self‐isolation is unnecessary, thereby unintentionally exposing others to the virus, including older people and others at high risk of morbidity and mortality. Infected people with false negative results are also less likely to seek RT‐PCR testing, especially as it now requires referral by a medical or nurse practitioner to a private pathology clinic.^3^ RT‐PCR tests can detect infections in their early stages, when antiviral treatments are most effective; not undergoing RT‐PCR testing could increase the risk that some people at greater risk of adverse outcomes forgo the possible benefits of early antiviral treatment.

It is reassuring that all eight studies reported high estimated test specificity (at least 97.9%); that is, the likelihood of false positive results is generally low and a positive result usually indicates a genuine infection, especially as tests are now generally motivated by suspicion of COVID‐19 (higher pre‐test probability).^29^

Our findings are similar to those of earlier systematic reviews, which found that the sensitivity of RATs varied substantially but that their specificity was consistently high.^2^, ^30^ The 2022 Cochrane review identified two studies that compared the effect of who interpreted RAT results on estimated sensitivity; each found that it was lower when interpreted by participants rather than by a medical practitioner.^2^ Other factors that can vary substantially between studies, including study design, participant characteristics, and test setting and conduct, can also influence estimated sensitivity. Consequently, indirect (between‐study) comparisons of test performance are difficult to interpret.^2^, ^30^ However, it is not clear on the TGA website that the sensitivity values listed are derived from different studies and should not be directly compared. It might be assumed that a “high sensitivity” test is more accurate than one with “acceptable sensitivity”. However, a Dutch within‐study comparison found that the Roche nasal self‐test was more sensitive than the All Test oral self‐test,^20^ for example, but the information on the TGA website could lead to the opposite conclusion (Roche: “acceptable sensitivity”; All Test: “high sensitivity”^5^).

### Limitations

Our review was based on searches in the comprehensive WHO and Cochrane COVID‐19 living data repositories, which employ robust search strategies to identify relevant studies.^9^, ^10^ Two reviewers were involved in each of the full‐text screening, data extraction, and risk of bias steps of the analysis. However, the identified studies assessed only eight TGA‐approved RATs, and we cannot comment on the diagnostic accuracy of other tests registered with the TGA. Since the time of our searches, the TGA has approved further tests and removed others from the self‐test list (78 TGA‐approved self‐tests were listed on 31 July 2023). Diagnostic accuracy studies for some newer tests may have been published after our search, or TGA approval may have been granted after our search. Risk of bias assessment for manufacturer‐reported studies was limited by the degree of information provided. Finally, the protocol for our systematic review was not registered, and the initial screening of titles and abstracts was undertaken by a single reviewer.

### Conclusion

Australia now relies on people isolating themselves and taking other precautionary measures for preventing and controlling COVID‐19, and self‐testing for infection plays a pivotal role in these decisions. However, evidence regarding the diagnostic accuracy of RATS when used as intended (unsupervised sample collection and interpretation) is limited. The substantial difference in sensitivity estimates between those supplied by manufacturers (and used to classify tests on the TGA website) and those from independent published studies is concerning. We hope that our findings will increase community awareness of the high risk of false negative results when using RATs for self‐testing and encourage people to not rule out infection solely on the basis of a RAT result if COVID‐19 is suspected. To improve the transparency of the evidence on its website, the TGA could require manufacturers to report their clinical studies according to the STARD guideline,^31^ facilitating independent risk of bias assessment with the QUADAS‐2 tool.^12^ It should also be made clear that the test sensitivity values listed on the TGA website are derived from different studies and should not be directly compared. Finally, we need better designed diagnostic accuracy studies of SARS‐CoV‐2 rapid antigen self‐tests.^32^

## Open access

Open access publishing facilitated by The University of Sydney, as part of the Wiley – The University of Sydney agreement via the Council of Australian University Librarians.

## Competing interests

No relevant disclosures.

## Supporting information


Supplementary methods and results
