# Supplementary material for: COVID‐19 rapid antigen tests approved for self‐testing in Australia: published diagnostic test accuracy studies and manufacturer‐supplied information. A systematic review
Source: Med J Aust. 2023 Oct 30;219(11):551–8. doi: 10.5694/mja2.52151 (PMC10952141; doi:10.5694/mja2.52151)
Supplement: Supplementary file 1 — Supplementary methods and results [file MJA2-219-551-s001.pdf]

MJA
The Medical Journal of Australia

Supporting Information

Supplementary methods and results

This appendix was part of the submitted manuscript and has been peer reviewed. It is posted as supplied by the authors.

Appendix to: Bell KJL, Li Y, Medcalf E, Ackermann D. COVID-19 rapid antigen tests approved for self-testing in Australia: published diagnostic test accuracy studies and manufacturer-supplied information. A systematic review. *Med J Aust* 2023; doi: 10.5694/mja2.52151.

Figure 1. PRISMA flow diagram for our systematic review

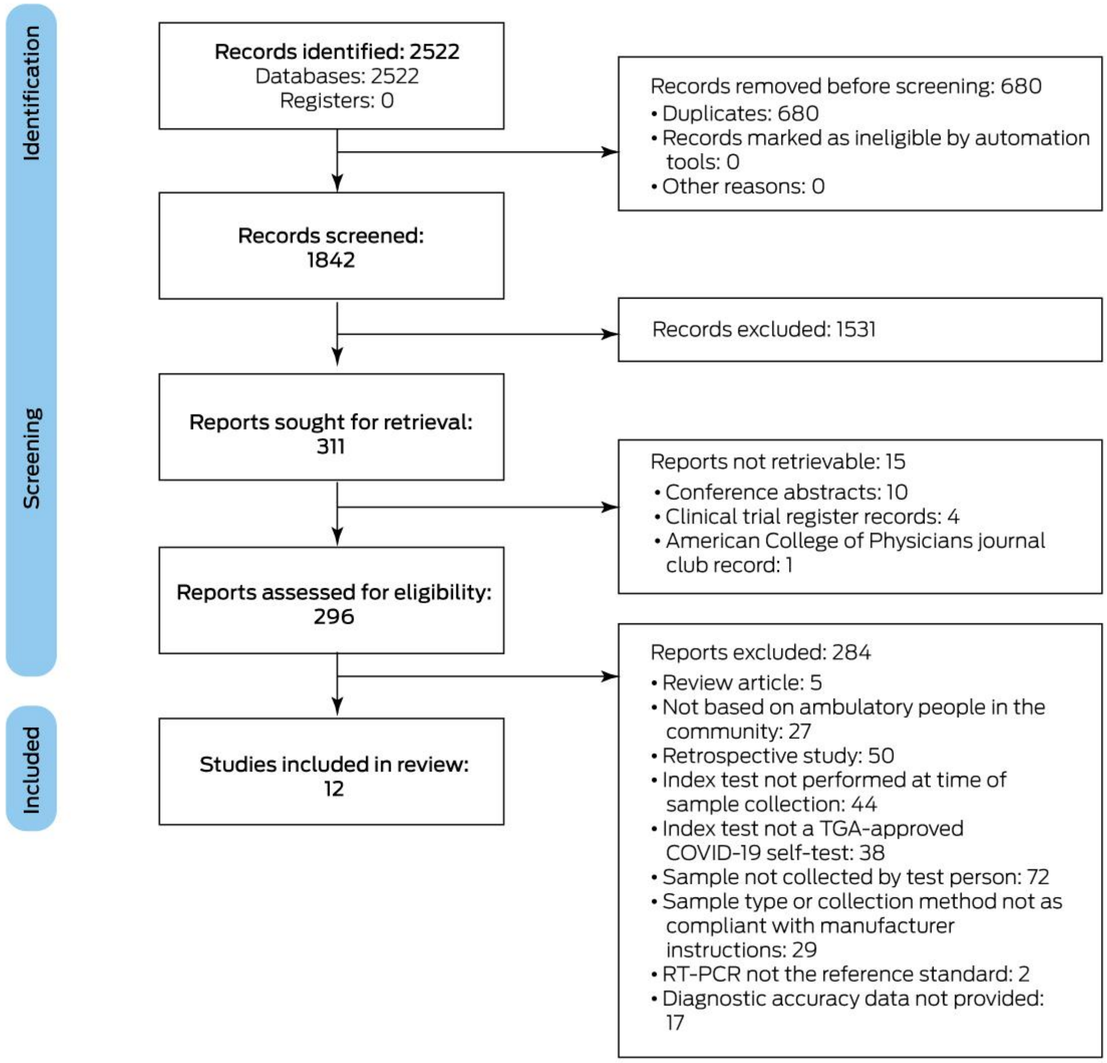

Figure 2. Risk of bias of the included published studies

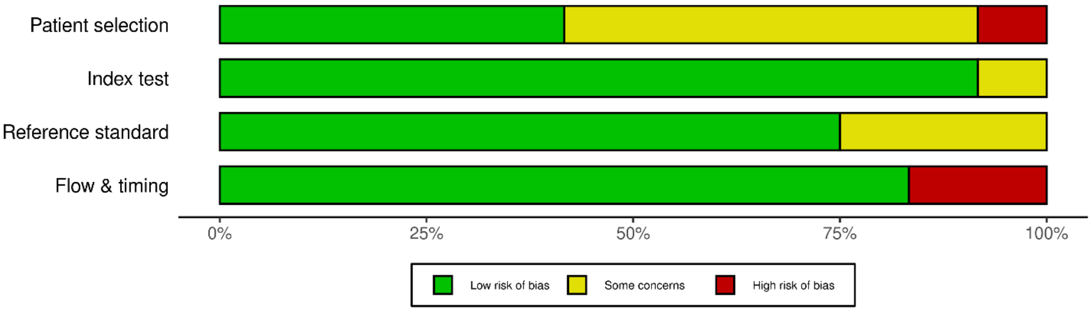

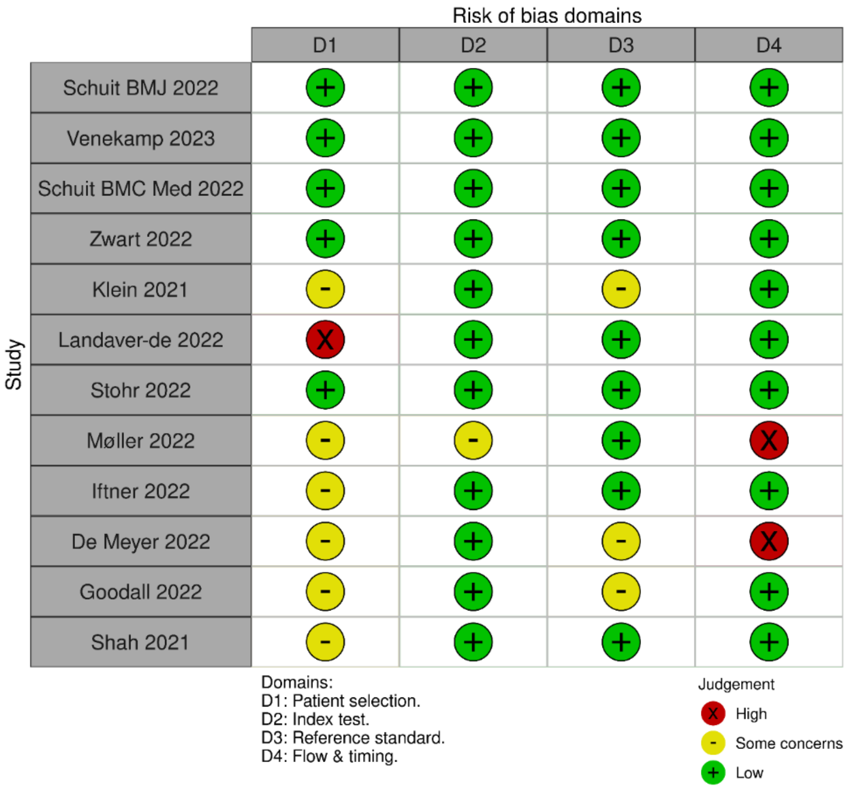

Figure 3. Risk of bias of manufacture supplied studies

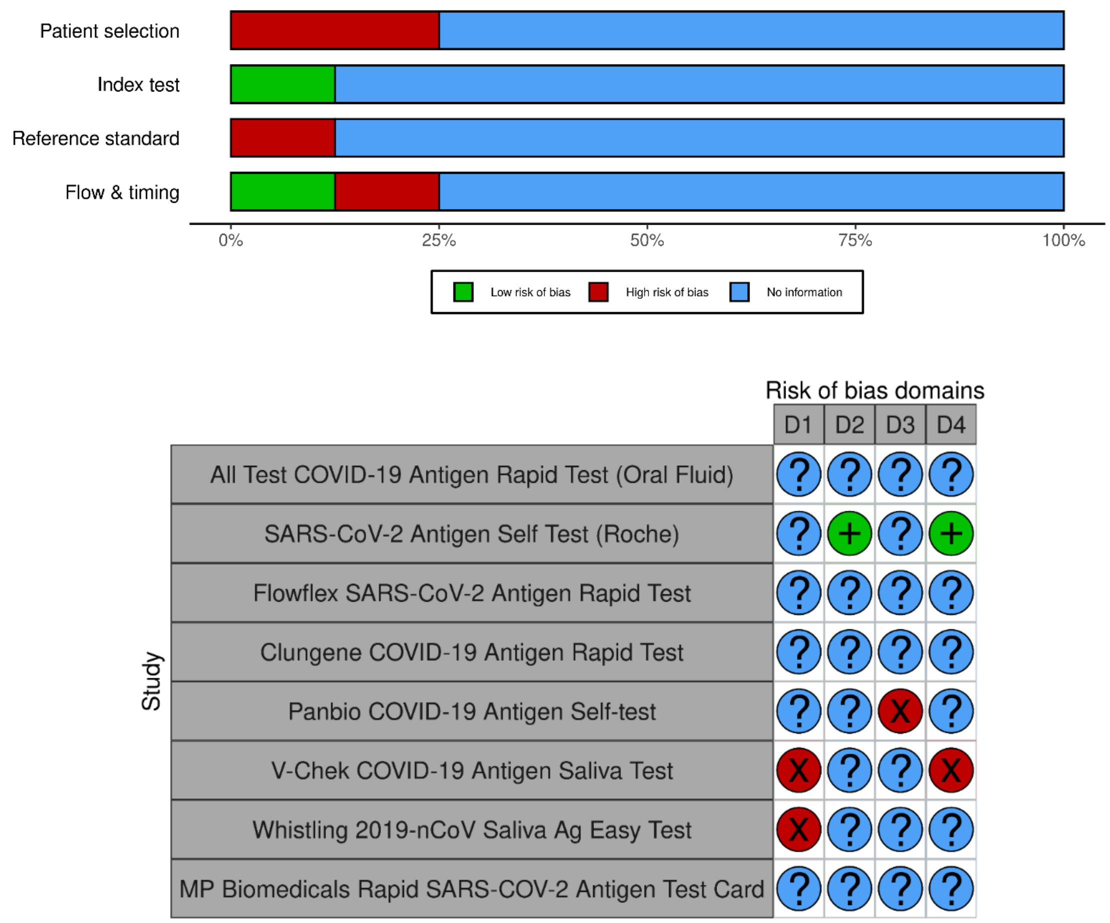

Test information:

1. All Test COVID-19 Antigen Rapid Test (Oral fluid): https://www.tga.gov.au/sites/default/files/covid-19-rapid-antigen-self-tests-are-approved-australia-oral-fluid-01.pdf

2. SARS-CoV-2 Antigen Self Test (Roche): https://www.tga.gov.au/sites/default/files/covid-19-rapid-antigen-self-tests-are-approved-australia-ifu-352250.pdf

3. Flowflex SARS-CoV-2 Antigen Rapid Test: https://www.tga.gov.au/sites/default/files/covid-19-rapid-antigen-self-tests-are-approved-australia-ifu-382031-02.pdf

4. MP Biomedicals Rapid SARS-COV-2 Antigen Test Card: https://www.tga.gov.au/sites/default/files/2022-08/covid-19-rapid-antigen-self-tests-are-approved-australia-ifu-01-373711.pdf

5. Clungene COVID-19 Antigen Rapid Test: https://www.tga.gov.au/sites/default/files/covid-19-rapid-antigen-self-tests-are-approved-australia-ifu-333341.pdf

6. Panbio COVID-19 Ag Rapid Test Device: https://www.tga.gov.au/sites/default/files/covid-19-rapid-antigen-self-tests-are-approved-australia-ifu-345192.pdf https://www.globalpointofcare.abbott/gb/en/product-details/panbio-covid-19-ag-antigen-test.html

7. BinaxNOW COVID-19 Antigen Card Self-Test: https://globalpointofcare.eifu.abbott/content/dam/ardx/eifu/iddm/scarborough/IN195150WEB%20v6.0%20BinaxNOW%20COVID-19%20Antigen%20Self%20Test%20Product%20Insert%20-%20English%20US.pdf

8. V-Chek COVID-19 Antigen Saliva Test: https://www.tga.gov.au/sites/default/files/covid-19-rapid-antigen-self-tests-are-approved-australia-ifu-374065.pdf

9. Whistling 2019-nCoV Saliva Ag EASY TEST: https://www.ridacom.com/en/products/view/7606 (Removed from TGA website as cancelled by sponsor (ARTG:391125)

10. Ecotest COVID-19 Antigen Saliva Test Kit: https://www.tga.gov.au/sites/default/files/covid-19-rapid-antigen-self-tests-are-approved-australia-ifu-372335.pdf

Table 1. Specificity of TGA-approved COVID-19 rapid antigen self-tests in published studies and in manufacturer supplied information

Author (publication year), country	Rapid antigen test (RAT)	Sample size*	RT-PCR-positive	Specificity reported by study	Specificity reported by manufacturer	Difference (percentage points)
**Unsupervised sample collection**						
Zwart (2022),[16] Netherlands	Roche SARS-CoV-2 Rapid Antigen Test (oropharyngeal and nasal)	2192	152	99.7%	100%	0.3
Stohr (2022),[17] Netherlands	Roche SARS-CoV-2 Antigen Self-Test (nasal)	1583	192	99.7%	100%	0.3
Møller (2022),[18] Denmark	Panbio COVID-19 Antigen Rapid Test Device (nasal)	388	0	99.7%	99.8%	0.1
Iftner (2022),[19] Germany	Clungene COVID-19 Antigen Rapid Test (nasal, researcher interpretation)	478	0	97.9%	100%	2.1
	Clungene COVID-19 Antigen Rapid Test (nasal, participant interpretation)	476	0	97.9%	100%	2.1
Schuit (2022),[20] Netherlands	All Test COVID-19 Antigen Rapid Test (oral fluid)	2803	182	99.0%	99.3%	0.3
	Roche SARS-CoV-2 Antigen Self-Test (nasal)	2819	180	99.5%	100%	0.5
Schuit (2022),[21] Netherlands	Flowflex SARS-CoV-2 Antigen Rapid Test (nasal)	341	145	98.5%	99.5%	1
	MP Biomedicals Rapid SARS-COV-2 Antigen Test Card (nasal)	581	169	99.3%	100%	0.7
	MP Biomedicals Rapid SARS-COV-2 Antigen Test Card (oropharyngeal and nasal)	255	88	98.2%	100%	1.8
Venekamp (2023),[22] Netherlands	Flowflex SARS-CoV-2 Antigen Rapid Test (nasal)	1229	193	99.8%	99.5%	-0.3
	MP Biomedicals Rapid SARS-COV-2 Antigen Test Card (nasal)	1027	115	99.8%	100%	0.2
**Supervised sample collection**						
Shah (2021),[23] USA	BinaxNOW COVID-19 Antigen Card Self-Test (nasal)	2110	334	99.6%	99.8%[2]	0.2
Klein (2021),[24] Germany	Panbio COVID-19 Antigen Rapid Test Device (nasopharyngeal)	290	45	99.2%	99.8%	0.6
DeMeyer (2022),[25] Belgium	V-Chek COVID-19 Antigen Saliva Test (oral)	50	13	100%	98.4%	-1.6
	Whistling test 2019-nCoV Saliva Ag Easy Test (oral)	102	55	100%	99.2%	-0.8
Goodall (2022),[26] Canada	Panbio COVID-19 Antigen Rapid Test Device (nasal)	825	62	100%	99.8%	-0.2
Landaverde (2022),[27] USA	BinaxNOW COVID-19 Antigen Card Self-Test‡ (nasal)	209	54	100%	99.8%[2]	-0.2

Notes:

1. Sample sizes exclude participants where RAT or RT-PCR result was unclear/inconclusive.
2. Manufacturer reported specificity is for Panbio COVID-19 Antigen Rapid Test Device.
3. TGA: Therapeutic Goods Authority; COVID-19: Coronavirus disease 2019; Self-RAT: rapid antigen self-test; PCR: Polymerase Chain Reaction; SARS-CoV-2: Severe acute respiratory syndrome coronavirus 2.

References

16 Zwart VF, van der Moeren, Stohr JJJM, et al. Performance of various lateral flow SARS-CoV-2 antigen self testing methods in healthcare workers: a multicenter study [preprint]. *medRxiv* 2022.01.28.22269783; 29 Jan 2022. https://doi.org/10.1101/2022.01.28.22269783 (viewed Sept 2023).
17 Stohr JJJM, Zwart VF, Goderski G, et al. Self-testing for the detection of SARS-CoV-2 infection with rapid antigen tests for people with suspected COVID-19 in the community. Clin Microbiol Infect 2022; 28: 695-700.
18 Møller IJB, Utke AR, Rysgaard UK, et al. Diagnostic performance, user acceptability, and safety of unsupervised SARS-CoV-2 rapid antigen-detecting tests performed at home. Int J Infect Dis 2022; 116: 358-364.
19 Iftner T, Iftner A, Pohle D, Martus P. Evaluation of the specificity and accuracy of SARS-CoV-2 rapid antigen self-tests compared to RT-PCR from 1015 asymptomatic volunteers. *medRxiv* 2022.02.11.22270873; 13 Feb 2022. https://doi.org/10.1101/2022.02.11.22270873 (viewed Sept 2023).
20 Schuit E, Venekamp RP, Veldhuijzen IK, et al. Head-to-head comparison of the accuracy of saliva and nasal rapid antigen SARS-CoV-2 self-testing: cross-sectional study. BMC Med 2022; 20: 406.
21 Schuit E, Venekamp RP, Hooft L, et al. Diagnostic accuracy of covid-19 rapid antigen tests with unsupervised self-sampling in people with symptoms in the omicron period: cross sectional study. BMJ 2022; 378: e071215.
22 Venekamp RP, Schuit E, Hooft L, et al. Diagnostic accuracy of SARS-CoV-2 rapid antigen self-tests in asymptomatic individuals in the omicron period: a cross-sectional study. Clin Microbiol Infect 2023; 29: 391.e1-391.e7.
23 Shah MM, Salvatore PP, Ford L, et al. Performance of repeat BinaxNOW severe acute respiratory syndrome coronavirus 2 antigen testing in a community setting, Wisconsin, November 2020 – December 2020. Clin Infect Dis 2021; 73 (Suppl 1): S54-S57.
24 Klein JAF, Krüger LJ, Tobian F, et al. Head-to-head performance comparison of self-collected nasal versus professional-collected nasopharyngeal swab for a WHO-listed SARS-CoV-2 antigen-detecting rapid diagnostic test. Med Microbiol Immunol 2021; 210: 181-186.
25 De Meyer J, Goris H, Mortelé O, et al. Evaluation of saliva as a matrix for RT-PCR analysis and two rapid antigen tests for the detection of SARS-CoV-2. Viruses 2022; 14: 1931.
26 Goodall BL, LeBlanc JJ, Hatchette TF, et al. Investigating the sensitivity of nasal or throat swabs: combination of both swabs increases the sensitivity of SARS-CoV-2 rapid antigen tests. Microbiol Spectr 2022; 10: e0021722.
27 Landaverde L, Turcinovic J, Doucette-Stamm L, et al. Comparison of BinaxNOW and SARS-CoV-2 qRT-PCR detection of the omicron variant from matched anterior nares swabs. Microbiol Spectr 2022; 10: e0130722.
